# Is intracrinology of endometriosis relevant in clinical practice? A systematic review on estrogen metabolism

**DOI:** 10.3389/fendo.2022.950866

**Published:** 2022-09-20

**Authors:** Antonio Mercorio, Pierluigi Giampaolino, Andrea Romano, Patrick Dällenbach, Nicola Pluchino

**Affiliations:** ^1^ Department of Pediatrics Gynecology and Obstetrics, Division of Gynecology, Geneva University Hospitals, Geneva, Switzerland; ^2^ Department of Public Health, School of Medicine, University of Naples Federico II, Naples, Italy; ^3^Obstetrics and Gynaecology Department, GROW - School for Oncology and Reproduction, Maastricht University, Maastricht, Netherlands

**Keywords:** endometriosis, estrogen, metabolism, pelvic pain, endometrium

## Abstract

Endometriosis is a chronic, multifactorial, estrogen-dependent disease. The abnormal endocrine microenvironment of endometriosis lesions is considered a main feature and multiple enzymatic pathways leading to local increased synthesis of estrogens have been identified. However, the relevance of intracrinology in clinical practice is still lacking. Medline, Embase, Scopus database were systematically searched for studies reporting on local estrogens metabolism of endometriotic lesions. The main enzymatic pathways involved in the intracrinology of endometriosis such as aromatase (CYP19A1), 17β-hydroxysteroid dehydrogenase (HSD17B) type 1, type 2 and type 5, steroid sulfatase (STS), estrogen sulfotransferase (SULT1E1) were assessed with a critical perspective on their role in disease endocrine phenotyping, drug resistance and as therapeutic targets. Overall, studies heterogeneity and missing clinical data affect the interpretation of the clinical role of these enzymes. Although the use of some drugs such as aromatase inhibitors has been proposed in clinical practice for two decades, their potential clinical value is still under investigation as well as their modality of administration. A closer look at new, more realistic drug targets is provided and discussed. Altered expression of these key enzymes in the lesions have far reaching implication in the development of new drugs aimed at decreasing local estrogenic activity with a minimal effect on gonadal function; however, given the complexity of the evaluation of the expression of the enzymes, multiple aspects still remains to be clarified.

**Systematic Review Registration:**

https://www.crd.york.ac.uk/prospero/display_record.php?ID=CRD42022311329, identifier CRD42022311329.

## Introduction

Endometriosis is described by the presence of endometrial-like tissue outside the uterus. It is defined as an estrogen-dependent, inflammatory, and chronic disease, often associated with pelvic pain and subfertility/infertility. The etiology remains uncertain and seems to be related to a complex interplay between family of genes associated with the immune system functioning, sex steroid hormone pathways (neuro)inflammation and environmental risk factors (1).

The estrogen-dependent nature of endometriosis is well known and clinically highlighted by the relapse of the disease in premenopausal women post discontinuation of anti-gonadotropic therapy, as well as in postmenopausal women following the administration of estrogen replacement therapy (2–4) Estrogens show direct cellular antiapoptotic and proliferative effects on the endometriosis lesion as well as promote the pro-inflammatory microenvironment, leading to the chronic nature of the disease (2).. Recent genomics, transcriptomics, and proteomics data, show that endometriosis tissue has a different hormonal environment (5). Increased estrogenic responsiveness, based on the aberrant number and ratio of ERalpha/ER beta (6), abnormal estrogen signaling, progesterone resistance have provided evidence that estrogen effects in normal endometrium do not fully replicate their action on endometriosis (7). Moreover, several studies have shown that endometriotic lesion represents a microenvironment where a multistep enzymatic process leads to an altered metabolism of DHEA or other androgen precursors into bioactive estrogens (**Table 1**). This mechanism which was called “intracrinology” was primarily defined by Fernand Labrie (8) as the combination of steroids that exhibit their action in the same cell without any pericellular space secretion. More recently, the term has been extended to the tissue microenvironment as the capacity to regulate tissue steroid concentrations from circulating precursors (9, 10).

**Table 1:** Estrogen concentrations in women with and without endometriosis.

Sample Type	Hormonal status	E2 (pg/ml)		E2/E1 ratio	
Healthy endometrium	Proliferative Secretory	Median 532 66	25/75% 334.7/736.0 52.5/100.8 ##	Median 8.34 0.69	25/75% 7.33/9.27 0.50/1.40###
Endometrium (Patients affected by endometriosis)	Proliferative Secretory	649.3 68.5	404.0/1168.7 25.0/157.3##	8.64 0.80	4.72/13.11 0.69/0.99###
Superficial endometriosis	Proliferative Secretory	238.0 176.0	78.9/397.0† 49.4/355.0	5.50 3.95	0.82/7.21 1.36/9.47
Ovarian endometriosis	Proliferative Secretory	3430.0 305.0	1809.7/21600.0* 199.0/758.0##	4.35 1.34	1.69/5.53 1.04/3.28#
Deep infiltrating endometriosis	Proliferative Secretory	112.0 25.0	53.9/162.0 25.0/117.3	1.06 2.00	0.60/2.60 * 1.44/4.77
Peritoneal fluid (22)	Proliferative Secretory	200 800

*P < 0.05 vs. eutopic endometrium within cycle phase. ^†^P < 0.05 vs. proliferative phase. #, ##, ###, P < 0.05, < 0.01, and < 0.001, respectively, vs. serum within the cycle phase. From Huhtinen K et al. Endometrial and endometriotic concentrations of estrone and estradiol are determined by local metabolism rather than circulating levels. J Clin Endocrinol Metab. 2012 Nov;97(11):4228-35.

The ability of peripheral tissues to use blood precursors and generate steroids has been quite well characterized for tissue remodeling and critical phases such as implantation, early pregnancy development and menstruation (11, 12).The evidence that intracrinology might play a role in the aberrant endocrine environment of endometriosis (13) have far-reaching implications for disease physiopathology, molecular characterization, and drug targeting. Although a large body of evidence in the last ten years supports the view that that endometriosis is a heterogeneous disease, molecular and endocrine clustering processes are just beginning to be understood. The macroscopic appearance of the disease poorly correlates with painful symptoms. Hormonal signatures have the potential to optimize disease characterization and management in the clinical setting. The present article aims to review the available literature investigating the association of intracrinology of endometriosis with clinical features of the disease. Current and ongoing clinical studies targeting intracrinology will be critically reviewed.

## Materials and methods

### Search strategy

Following protocol registration with PROSPERO (Registration number CRD42022311329) a systematic review was conducted according to Preferred Reporting Items for Systematic Reviews (PRISMA) guidelines. No approval from the Institutional Research Ethics Committee was sought owing to the nature of this work. The following search query was submitted throug Medline, Embase and Scoups database: (“oestrogen”[All Fields] OR ′′estrogens”[MeSH Terms] AND (′′metabolism”[All Fields] OR ′′metabolism”[MeSH Terms] OR ′′metabolic networks and pathways”[MeSH Terms] AND (“endometriosis”[MeSH Terms] OR (“endometriosis”[All Fields]). In addition, Embase and Medline were searched with broader terms: endometriosis AND estrogen metabolism AND steroid sulfatase; endometriosis AND estrogen metabolism AND 17beta hydroxysteroid dehydrogenase; endometriosis AND estrogen metabolism AND estrogen sulfotransferase.

### Study selection

We limited the search to publications in English and excluded article published earlier than 1995. Potentially eligible studies were retrieved in full text for the assessment of their eligibility. Study selection was conducted independently by two reviewers (AM and NP) with disagreement resolved by consensus. We included all studies that satisfied the following eligibility criteria, i) They were human based studies. Studies on animal models and *in vitro* studies investigating human tissue derived cell lines were excluded, ii) They presented original research data, iii) Participants had endometriosis confirmed by histology. In order to highlight the association of the enzymes involved in the intracrinology of endometriosis and the clinical features of the disease, we decided to focus preferentially on their protein expressions levels which reflects gene function more directly than mRNA and is more directly related to phenotype; however, due to the paucity of data present in literature this focused analysis was possible only for the “aromatase” enzyme.

### Data extraction and analysis

A standardized critical appraisal and data extraction tool was generated using criteria from CASP (Critical Appraisal Skills Programme) and statements, PRISMA, STARD (Standards for Reporting Diagnostic accuracy studies) and STROBE (Strengthening The Reporting of Observational Studies in Epidemiology) as appropriate. Two reviewers independently appraised the articles and extracted data (AM and NP).

### Risk of bias

To reduce selection bias, the abstracts and full text papers were evaluated by masking the authors as far as possible and by basing decisions regarding relevance and eligibility on the independent appraisal by two reviewers. Bias in studies included was assessed independently by the two reviewers.

## Results

### Study selection

**Figure 1:**
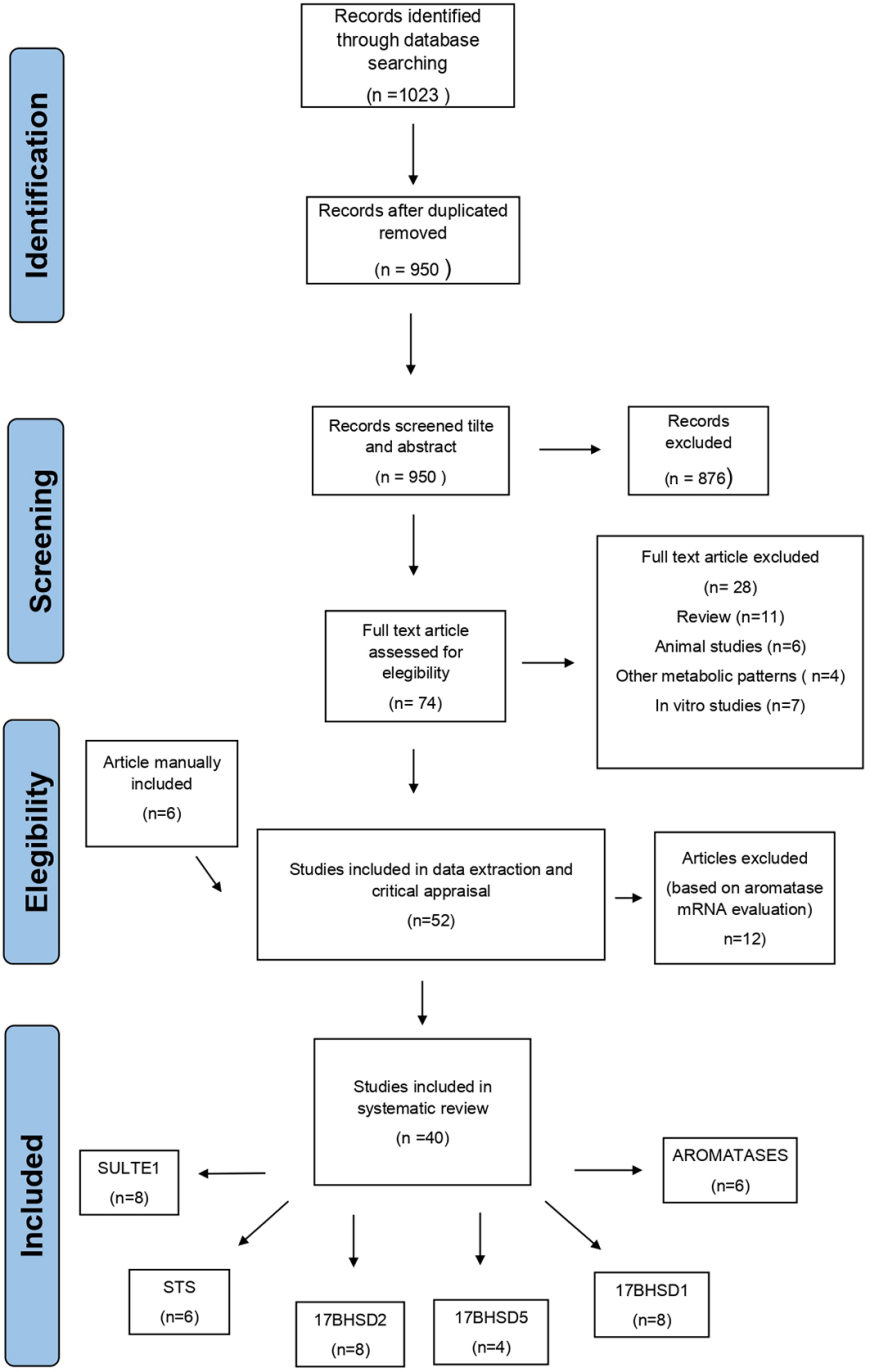
Flow diagram of the systematic search strategy on published studies reporting about the expression of the enzymes involved in the intracrinology of endometriosis.

Total articles retrieved after duplicated removal were 1023. Analysis of the titles and abstract led to the removal of 949 papers, including repeated hits and articles based on disease other than endometriosis or full text in English not available. Out of the 74 articles remained for full-text analysis, 28 were excluded because they did not provide original results (i.e. reviews) or they referred to preclinical data. References in the selected articles were controlled for missing inclusions and six articles were included manually (**Figure 1**). In the end a total of 40 articles were included in this systematic review.

### Characteristics of the included studies

**Figure 2:**
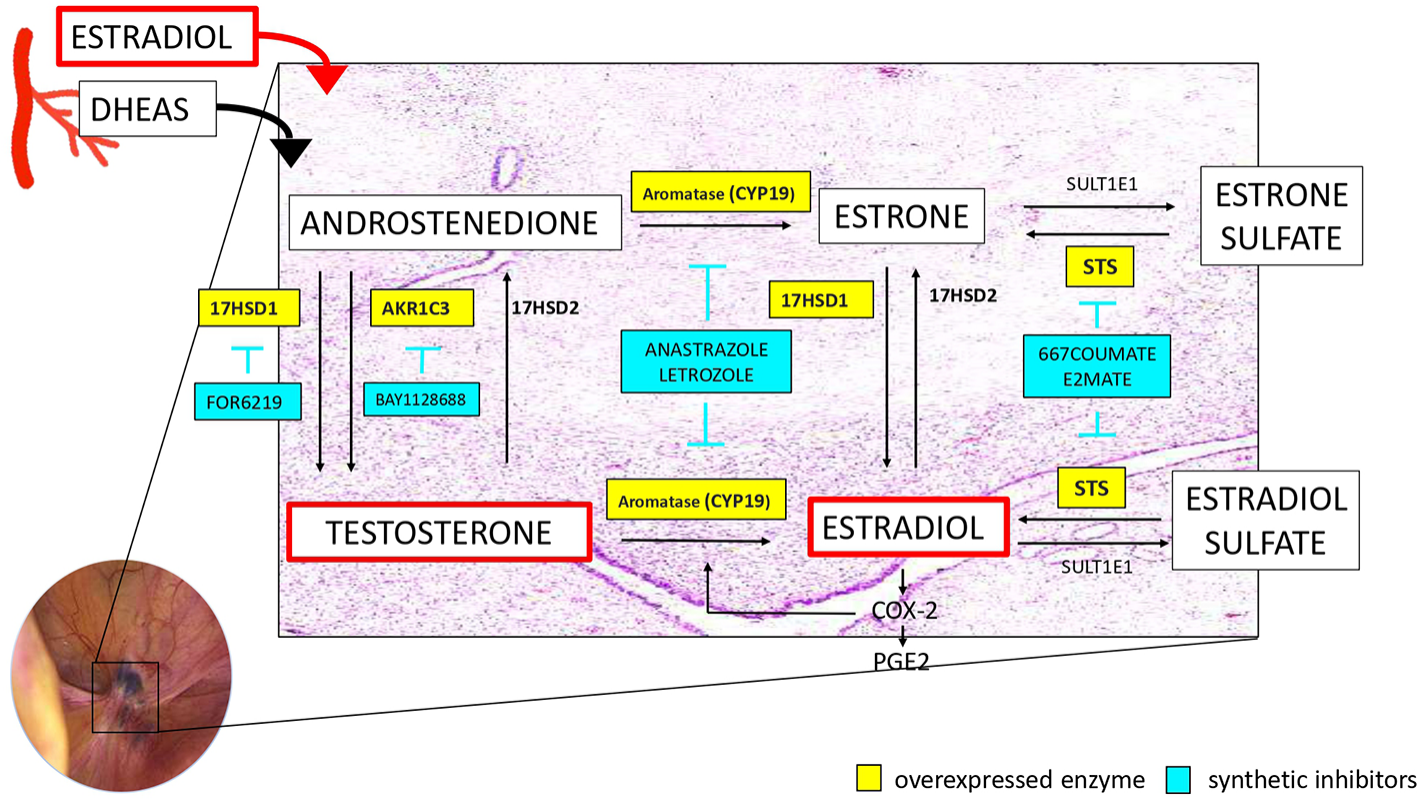
Schematic representation of the intracrine estrogen metabolism with the additional data on the overexpressed enzymes and their synthetic inhibitors.

In the following sections, studies evaluating the expressions of the enzymes controlling the synthesis/inactivation of estrogens in endometriosis are examined with respect to clinical stage of disease, severity of symptoms and disease localization. Analysis of targeted enzyme therapy and current hormonal treatments effect on intracrinology regulation are provided. Four are the principal pathways that have received more attention and that likely contribute more than others to aberrant endocrine regulation: aromatase,17β-hydroxysteroid dehydrogenase, sulfatase and sulfotransferase (**Figure 2**).

## Aromatase

Aromatase has been extensively investigated in endometriosis. However, findings are still conflicting. Aromatase is encoded by the CYP19A1 gene, a member of the cytochrome P450 superfamily, which consists of monooxygenases that catalyze several reactions involved in steroidogenesis. It is physiologically expressed in growing ovarian follicles by granulosa cells, promoting the conversion of androstenedione (A4) and testosterone (T) to estrone (E1) and estradiol (E2), respectively. In the last ten years, it has been proposed that endometriotic tissue features different aromatase expression compared to normal endometrium, which is responsible for the local production of estradiol and in turn stimulates the production of the cyclooxygenase type 2 (COX-2) enzyme, resulting in elevated levels of prostaglandin E2 (PGE2), which is a potent stimulator of aromatase activity in endometriosis (14). Nevertheless, it should be taken into account that immunohistochemistry is a semi-quantitative method which does not consent the actual “quantification” and cannot determine the activity of an enzyme but only detect its presence. This positive feedback produces a local, continuous stream of estrogen and PGE2 in endometriotic tissue, supporting a loop between a hyperestrogenic environment, inflammation and cell proliferation (15, 16). *In vitro* and *in vivo* studies clearly show the expression of aromatase in stromal and epithelial cells within endometriotic lesions. *In vitro*, mechanistic models support its crucial role. However, the full picture of endometriotic aromatase in the clinical setting has not been fully clarified.

### Aromatase expression in human tissue

To date, most investigations have been performed at the mRNA level or using Western blotting. There is a paucity of data utilizing tissue staining techniques, and the results are conflicting. Although mRNA expression is informative of protein expression, mRNA copies do not necessarily reflect the level of functional protein: posttranscriptional and posttranslational regulation induces functionally important changes that cannot be seen at the mRNA level (17). Therefore, we focus mainly on protein expression, which reflects gene function more directly than mRNA and is more directly related to phenotype (**Table 2**). Based on our research criteria, only six studies evaluated aromatase expression at the protein level using immunohistochemistry. The level of staining differs among studies in relation to the type of endometriosis, number of positive patients and correlation with clinical symptoms.

**Table 2:** Expression (protein levels), enzymatic activity of aromatase in endometriotic lesions and correlation with disease characteristics, eutopic and healty endometrium.

Authors	Patients with endometriosis (n)	Method (Antibody)	Expressionor activity	Lesion localization	Severity of symptoms	Stage of disease	Eutopic endometrial tissue	Control
Hudelist et al. 2007 (18)	35	IHC (Harada)	32/35	* Mixed. * No Correlation	No correlation	* Moderate and severe (ASRM) * No correlation	Positive	Negative
Acien et al. 2007 (19)	62	IHC (DPC Biermann)	38/62	* Mixed. * Positive correlation with ovarian lesions (especially bilateral)	Positive correlation with moderate to severe chronic pelvic pain	* Moderate and severe (ASRM) * No correlation	Negative	Negative
Colette et al. 2009 (20)	127	IHC (Acris. Serotec)	No expression	* Mixed	–	–	Negative	Negative
Maia et al. 2012 (21)	92	IHC (Serotec)	66/92	* Endometrial biopsy	Positive correlation with dysmenorrhea (even in patients free from endometriosis)	* ASRM I to IV * Positive correlation with stage of disease	Positive	Negative
Pluchino et al 2020 (6)	83	IRS (Harada)	–	* Deep endometriosis involving the pouch of the Douglas	Positive correlation with moderate to severe deep dyspareunia	* AFS III–IV	–	–

AFS, American Fertility Society; ASRM, American Society for Reproductive Medicine; HPLC high performance liquid chromatography; IHC, immunohistochemistry; IRS, immunoreactive score.

In two cohorts of mixed lesions (ovary, peritoneal and deep implants), positive immunohistochemical expression of aromatase was found in only 38/62 women (61.3% of patients with histologically confirmed endometriosis) (21) and in 32/35 patients with mild to moderate endometriosis according to the revised American Fertility Society (AFS) classification (18). These patients showed multiple endometriomas, frequently bilateral, and more moderate-to-severe chronic pelvic pain. Eutopic endometrium often stained aromatase in endometriosis patients (18, 21), being detectable by IHC in 66/92 patients but also in the endometrium of 13/14 (95%) patients with severe dysmenorrhea but free from endometriosis. Interesting in asymptomatic women (control group) aromatase expression was not detected (18). This finding suggests that functional endometrial changes leading to an increase in aromatase expression may therefore precede the development of endometriosis and have a positive correlation with dysmenorrhea severity.

In a recent study, patients experiencing moderate to severe deep dyspareunia showed higher epithelial aromatase in deep lesions involving the pouch of the Douglas than patients experiencing absence to mild dyspareunia (8). However, in 209 samples of mixed-type endometriosis (peritoneal, ovarian and rectovaginal lesions), aromatase expression was not detected in either ectopic or eutopic endometrial tissue, although there was no mention of pain symptoms (23) or even stage of disease (20).

Unfortunately, conflicting data and methodological concerns affect the interpretation of the role of this enzyme in endometriotic implants, and consistent proof of aromatase expression is still lacking. Some of the variations in the reported patterns of expression may relate to the use of different antibodies, analysis of eutopic endometrium at different stages of the cycle, the type of lesion or the inflammatory status. Moreover, fragments of ovarian tissue may be included in the histological sections of specimens of ovarian endometriosis, affecting the accuracy of the evaluation.

### Aromatase inhibitors (AIs)

Despite the paucity of human data, aromatase blockade has been proposed as a treatment for endometriosis since its first successful use in a postmenopausal woman with aggressive and refractory endometriosis (24). Thereafter, different studies, published over the last twenty years, have evaluated the efficacy of AI treatment alone or combined with other hormonal therapies. Selective third-generation AIs, letrozole and anastrozole, have been found to have better efficacy and tolerability than earlier agents (25). Seven clinical trials have compared the use of these drugs with other treatments for endometriosis (**Table 3**). Aromatase inhibitors, whenever given in combination with progestins, GnRHa or administered alone, showed greater results in terms of pain reduction than conventional hormonal treatments. Anastrozole (1 mg/day), administered in conjunction with goserelin for 6 months in patients with severe baseline endometriosis (rASRM score >40), showed greater improvement in pain Total Pelvic Symptom Score (TPSS) than patients using goserelin alone (ΔTPSS-P baseline -24 months PMT 5.0 vs. 3.3 p<0.0001) (26). The efficacy of AIs combined with norethisterone acetate (NETA) (letrozole 2.5 mg/day plus norethisterone acetate 2.5 mg/day) versus NETA alone (2.5 mg/day) has been specifically evaluated in rectovaginal endometriosis (27). Significantly lower dyspareunia and chronic pelvic pain were reported in the group receiving the double drug regimen after 3 months. Letrozole was also investigated as single molecule in a randomized clinical trial based demonstrating the lowest mean chronic pelvic pain at 5 months compared to danazol (600 mg/day) and placebo. No data, however, are available regarding the stage, type, and localization of disease (28). Four additional studies investigating more than 200 patients showed comparable results in terms of pain reduction between AIs vs. GnRHa or AI plus NETA vs. NETA alone or AI plus GnRH (29–32), making less clear the clinical added value of AI over conventional, first-line, treatments. The onset of recurrence of symptoms during follow-up was always comparable among all studies. Adding AI to current hormonal treatment does not offer advantages over the single drug, as control group. Only one study, showed a longer interval before recurrence of symptoms during the 24-month follow-up: recurrence was registered in only 3/40 (8%) of patients in the combined arm (AI + goserelin) compared to 14/40 (35%) in the goserelin-only arm (26). Regarding side effects, AIs in association with progestins did not seem to have a more detrimental impact than progestin alone in two trials (30, 32). However, in Ferrero et al., a high incidence of adverse events in patients taking letrozole plus norethisterone acetate was registered (two patients interrupted the treatment because of severe joint pain, others had severe migraine attacks, myalgia and breakthrough bleeding) (27). As a consequence, “global degree satisfaction” was not different among groups despite a better pain reduction in patients receiving AI plus progestins. When AI was associated with GnRHa, a higher rate of side effects was found, and Ferrero et al. were forced to end the study preterm due to the high incidence of adverse effects (arthralgia, decreased libido, hot flashes) (30). This supports the hypothesis that cotreatment with progestins is more accepted than cotreatment with gonadotropin-releasing hormone analogs. In terms of bone mineral density, no significant change were reported when AIs were combined with NETA (27). In contrast, when associated with GnRH, AIs resulted in a significant decrease in the mineral bone density at 6 months (26, 30); however, none of these patients fell into the category of osteopenia and, furthermore, this reduction was not confirmed at 2 years of treatment withdrawal (26).

**Table 3:** Clinical trials comparing AIs (alone or combined) with other treatments in women with endometriosis.

Authors	Type of study	Treatments	Length of treatment/Follow Up(months)	Stageof disease	Type of lesion	Recurrence	Evaluation of pain	Adverseeffects	Lesion reduction(US)	Bone loss(BMD)
Soysal et al. 2004 (26)	Patients: 40 vs 40 Double arm blind RND.	Anastrazole 1mg/day + Goserelin 3.6mg/4w vs Placebo + Goserelin 3.6mg/4w	6/24	Severe endometriosis (ASRM score >40)	–	Later and less recurrence rate of symptoms	Greater reduction for the whole follow up (TPSS)	No difference at 24 weeks “menopausal quality of life score”	–	Greater bone loss (24 weeks) No difference (24 months)
Ferrero et al. 2009 (27)	Patients: 41 vs 41 Double arm OL non RND	Letrozole 2.5mg/d + NETA 2.5mg/d Vs NETA	6/12	–	Rectovaginal nodules	Comparable recurrence	Greater reduction for chronic pelvic pain and dyspareunia (VAS)	More frequent and no difference in global treatment satisfaction	–	No difference
Roghaei et al. 2010 (28)	Patients: 38 vs 37vs 31 Triple arm OL RND	Letrozole 2.5mg Vs Danazol 600mg Vs Placebo	6/6	–	–	–	Greater reduction of chronic pelvic pain and dyspareunia at 5 months (VAS)	Less frequent and severe	–	–
Alborzi et al. 2011 (29)	Patients: 47vs 40vs 57 Triple arm OL RND	Letrozole 2.5mg Vs Triptorelin 3.75mg/4w Vs No medication	2/12	AFS I - IV	–	Comparable recurrence	Comparable reduction (VAS)	More functional cysts formation	–	–
Ferrero et al. 2011 (30)	Patients 17 vs18 Double arm OL RND	Letrozole 2.5mg/d + NETA 2.5mg Vs Letrozole 2.5mg/d + Triptorelin 11.25mg/3m	6/6	–	Rectovaginal nodules	–	Comparable reduction (VAS)	More adverse effect and more patients left the study in Letrozole + triptorelin group	Greater reduction In Letrozole + Triptorelin group	Greater bone loss in Letrozolo+ Triptorelin group
Ferrero et al 2013 (31)	Patients 18 vs10 vs 8 vs 26 vs 30 Multiple arm OL not RND	NETA 2.5mg/d Vs Triptorelin 11.25/3m + tibolone 2.5mg/d Vs Letrozole 2.5mg/d + NETA 2.5mg/d Vs desogestrel 0.075mg/d Vs EE0.02mg + Desogestrel 0.15 mg	12/12	–	Rectovaginal nodules	–	–	Comparable	Significant and comparable reduction for all treatments	–
Ferrero et al 2014 (32)	Patients: 20vs20 Double arm OL non RND	Letrozole 2.5mg/d + NETA 2.5mg/d Vs NETA 2.5mg/d	6/12	–	Ovarian endometrioma	Comparable recurrence	Comparable reduction (VAS)	Comparable with no difference in global treatment satisfaction	Significant and comparable reduction (6 months) return to baseline dimensions (6months after treatment)	–

AFS,American Fertility Society; ASRM, American Society for Reproductive Medicine; TPSS, Total pelvic symptom score;VAS, visual analogue scale; NETA, norethisterone acetate; RND randomized; OL, open label; US, ultrasound; BMD, bone mass density.

Three trials evaluated whether AIs were superior to other hormonal treatments for the regression of endometriosis lesions (30–32). The combination of letrozole plus NETA showed a greater reduction in endometrioma at 6 months of treatment than the administration of Neta alone (-74.4± 4.2% vs. -46.8±3.8%). Regrettably, no patients reported a complete regression of cysts, and endometriomas usually regrew after treatment discontinuation (32). Two studies confirm the reduction of the size of rectovaginal lesions (30, 31). In a multiple arms trial, letrozole 2.5 mg/d + NETA 2.5 mg/d was compared with other first-line regimens and no difference of volume reduction was recorded (31). When letrozole is administered in combination with triptorelin demonstrated a significant reduction in the volume of the endometriotic nodules after 6 months (16.1 cm3 ± 10.0% vs. 10.2 cm3 ± 6.3%; p = 0.048) in comparison to NETA alone (30).

The heterogeneity of the current literature limits the evaluation of the potential advantages provided by the use of AIs for the treatment of endometriosis as well as the assessment of side effects: severity and type of the disease are often underreported, and follow-up of pain symptoms is not long enough to make consistent conclusions regarding their implementation in clinical practice. Based on current recommendations, AIs should be now offered only in case of surgical and other hormonal therapies failure (33). To minimize the impact on systemic estrogen levels, administration of an aromatase inhibitor (AI) *via* an intravaginal ring (IVR) has been proposed. This approach offers the advantage of providing sustained and controlled drug release and requires a lower dose to achieve equivalent pharmacodynamic efficacy compared to oral administration (it avoids the first-pass effect of the liver) (34). Phase I and Phase II studies on an IVR containing a combination of anastrazole and levonorgestrel gave reassuring results in terms of safety and tolerability at different doses as well as contraceptive efficacy (35, 36). However, the clinical effect of this combination is still unknown. A pilot study demonstrated modest efficacy following administration of vaginal anastrozole (0.25 mg/d) for 6 months offered to 10 women suffering from rectovaginal endometriosis: although a small, statistically significant improvement in dysmenorrhea was observed, chronic pelvic pain, dyspareunia and rectovaginal lesion size remained unchanged. The inefficacy of the therapy could be attributed to inadequate dose exposure given the absence of a reduction in circulating E2 (37). Endometriosis-targeted inhibition of local aromatase, despite its promising potential, has recently been discredited, using a new chick embryo allantoic membrane (CAM) model incorporating xenografted human endometriosis cysts: in this recent study was shown that topical treatment with anastrozole reduced the size of the lesions, corroborating the presence of aromatase activity in endometriotic tissue. However, when systemic estrogens reached the grafted endometriotic tissue, the effect of the local inhibition of aromatase by anastrozole was blunted. This finding supports the speculation that endometriosis aromatase cannot be a drug target without the inhibition of systemic estrogen synthesis (38).

## 17β-Hydroxysteroid dehydrogenase (HSD17B)

17β-Hydroxysteroid dehydrogenase (HSD17B) isozymes are a group of alcohol oxidoreductases, of which several isoforms are expressed in human tissues. Of particular interest is the activity of HSD17B1, which is responsible for the production of testosterone (T) and 17 beta estradiol (E2) from weak androstenedione (A4) and estrone (E1), respectively, and HSD17B2, which, in contrast, catalyzes the opposite reaction, metabolizing E2 to the less active E1. These pathways are believed to be involved in the abnormal ratio E2/E1 in the ectopic endometrium. Interesting data on the activity of these enzymes in endometriosis comes from Delvoux et al: in this study, endometriotic tissues of 14 women affected by moderate to severe endometriosis (endometrioma, deep infiltrative and superficial peritoneal endometriosis) showed a marked increase in HSD17B1 activity, leading to higher estradiol (E2) production than normal endometrial tissue (23). Conversely, the activity of the enzymes responsible for the oxidation of 17-estradiol into the less active estrone was significantly lower. Therefore, the net balance between oxidization and reduction favored the production of 17-beta E2 in ectopic tissues compared with endometrial tissues of healthy patients.

### HSD17B1 expression in human tissue

Despite this premise, discordant results arise from studies evaluating HSD17B1 expression (**Table 4**). At the protein level, there was no evidence of HSD17B1 hyperexpression in endometriosis tissue (41, 45). In 14 samples of rectovaginal endometriosis, HSD17B1 protein levels were found to be even lower than those in normal endometrium. This result diverges from HSD17B1 mRNA level, which was higher than in controls, raising the hypothesis that the expression of the HSD17B1 gene may therefore not necessarily reflect changes at the functional protein level (41). Delvoux et al. and found a greater than 6000-fold increase in HSD17B1 mRNA expression in endometriosis (Stade IV rASRM, mixed lesions) compared to eutopic tissue (46). In addition, inhibition of HSD17B1 by a specific inhibitor (3-[15b-estronyl]-N-(5-methyl-thiazol-2-yl)-propionamide) was achieved, decreasing the production of 17-estradiol by at least 85% in 70% of patient biopsies tested ex-vivo. Three additional studies (a total of 101 patients were evaluated), upregulation of the HSD17B1 enzyme was confirmed only in ovarian endometriosis (42–44). Interestingly, data from two studies (39, 45) are in contrast with the abovementioned results and no difference of HSD17B1mRNA between endometriosis (each type) and controls (eutopic endometrium in patients without endometriosis) were detected.

**Table 4:** 17BHSD type 1, type 2 mRNA and protein expression in endometriotic tissue.

Authors	Patient with endometriosis	Method	Lesion localization	Stage of disease	17 BHSD type 1 expression (compared to healthy endometrial tissue)		17 BHSD type 2 expression (compared to healthy endometrial tissue)	
					mRNA	Protein (Ab)	mRNA	Protein (Ab)
Zeitoun et al.1998 (39)	14	Northern Blot IHC	Extraovarian endometriotic implants	–	≈	-	↓	↓ (C2-12)
Matsuzaki et al. 2006 (40)	16	Q-PCR	Rectovaginal	–	-	-	–	-
Dassen et al.2007 (41)	14	Q-PCR IHC	Rectovaginal	–	↑	↓ (Pineda)	–	-
Smuc et al. 2007 (42)	16	Q-PCR	Endometrioma	Moderate and severe (ASRM)	↑	-	≈	-
Smuc et al. 2009 (43)	24	Q-PCR	Endometrioma	–	↑	-	≈	-
Huhtinen et al. 2012 (44)	60	Q-PCR	Peritoneal Ovarian Deep endometriosis	–	↑ in endometrioma	-	↓	-
Colette et al. 2013 (45)	79	Q-PCR IHC	Peritoneal, ovarian, rectovaginal	–	≈	≈ (Novocastra)	↓ in endometrioma. (Protein and rectovaginal Tech group) lesion	≈
Delvoux et al. 2014 (46)	29	Q-PCR HPLC	Peritoneal, ovarian, rectovaginal	Moderate and severe (ASRM)	↑ Inhibitor tested lead to decreased production of 17 beta estradiol	-	≈	-

Ab, Antibodies: ASRM, American Society for Reproductive Medicine; HPLC high performance liquid chromatography; IHC, immunohistochemistry; Q-PCR, Quantitative-Polymerase chain reaction, ↑/↓ statistically significant results: ≈, no difference.

### HSD17B2 expression on human tissue

The HSD17B2 enzyme is involved in the oxidative reaction and is responsible for the metabolization of potent E2 to the less active E1. The evaluation of HSD17B2 at the protein level is scanty and based only on two studies with conflicting results (39, 42–46), showing lower expression (39) or no changes (45) in endometriotic tissues (mixed lesions). Concerning mRNA expression, HSD17B2 appeared to be reduced in all types of endometriotic lesions compared to controls (39, 44, 46). Deep endometriosis shows undetectable levels of HSD17B2 mRNA type 2 in 50% of patients (8/16), and low levels were found in the remaining patients (40). In the study of Colette et al., where the protein level did not differ between samples, lower mRNA expression in rectovaginal and ovarian lesions but not in superficial peritoneal lesions was observed (45). Surprisingly, in endometrioma tissues evaluated by Smuc et al. (42, 43), no statistically significant difference was found with respect to healthy patients.

The heterogeneity, in terms of the method used to evaluate mRNA and protein expression, of the abovementioned studies prevent a proper comparison. Moreover, from a clinical perspective, given the lack of data regarding the stage of disease (only Delvoux et al. specified the characteristics of their patients) and the correlation with the severity of symptoms, it is difficult to estimate which patient can really benefit from future targeted enzyme therapy.

### HSD17B5 expression on human tissue

Worth of mention for its ability to influence multiple signaling pathways in endometriosis is 17β-hydroxysteroid dehydrogenase type 5 (17BHSD5) also known as AKR1C3. This steroidogenic enzyme can function as a PGF2α synthases, increasing the concentration of prostaglandins in peritoneal fluid (47), and catalyze the reduction of progesterone to the less active 20α-hydroxyprogesterone, leading to a defective progesterone action and contributing to the progesterone-resistant state (48). Concerning its roles in androgen and estrogen biosynthesis it has a very high catalytic efficiency for the conversion of androstenedione to testosterone which may finally act as a substrate for aromatase, having thus an indirect role in estradiol formation (49). In term of protein levels using IHC, two studies reported, the presence of AKR1C3 in endometriomas (43) and peritoneal endometriotic lesions (50). However, when scoring of AKR1C3 staining was performed, no significant differences in endometriosis lesions (ovarian endometriomas) compared to the endometrium of control patients were revealed (47, 51). Data provided on mRNA levels in endometriotic tissue are even more discordant. In a study evaluating 24 samples of ovarian endometrioma mRNA reported a higher expression respect to controls (43), whereas in a study reporting the analysis of 31 ovarian endometrioma only a slight difference was observed (51). Furthermore no data on pain symptoms or endometriosis stage are reported, making difficult further comparison. Interesting results from a study where peritoneal endometriosis samples were classified according to menstrual cycle phase (50): increased expression of AKR1C3 was observed in women with disease stages I–II and during the proliferative phase of the menstrual cycle. However, when all type of endo are analyzed together, only minor differences of mRNA expression (44) were detected. As consequence, further evidence to confirm the clinical relevance of AKR1C3 as a target in endometriosis are then needed.

### HSD17B inhibitors

Some inhibitors of HSD17B1 were developed in the past to target the biosynthesis of bioactive E2 in breast cancer (52). However, only a few compounds have been applied *in vivo* (53). Differences between enzymes in humans and other species are one of the main reasons that preclinical *in vivo* evaluation has been hindered.

A novel HSD17B1 inhibitor, FOR-6219, recently successfully completed a Phase 1a study in which the safety, tolerability, and pharmacokinetics of single and multiple ascending doses in 36 healthy postmenopausal women were investigated (NCT03709420). In Phase 1b, 36 premenopausal healthy women were investigated to expand the safety data and explore secondary outcome measures; interestingly, these women continued to experience normal ovulatory menstrual cycles (Report No.: NCT03709420. Available from: https://clinicaltrials.gov/ct2/show/NCT03709420).

Forendo Pharma is now planning a Phase 2 program including endometriosis patients in the US (available from: https://forendo.com/forendo-pharma-successfully-completes-phase-1-studies-of-for-6219-in-endometriosis-aiming-to-advance-program-into-phase-2-clinical-studies/). A steroidal inhibitor of AKR1C3, BAY1128688, was tested in a phase I clinical trial (NCT02434640) to investigate its safety, tolerability and pharmacokinetics in healthy women, and it appeared to be well tolerated up to a high dose of 60 mg twice per day. In a phase II clinical trial (NCT03373422) designed to evaluate the reduction of pain and the incidence of adverse events, it was planned to treat symptomatic women with endometriosis over a 12-week treatment period. Unfortunately, the trial was stopped in advance due to hepatotoxicity. A recent review, however, concluded that hepatotoxic effects can be compound-related, and AKR1C3 should not be precluded as a potential target (48). The development of other drugs targeting this enzyme is ongoing.

## Sulfatase and sulfotransferase

Sulfatase (STS) is an enzyme involved in another critical alternative pathway that contributes to the increased bioavailability of regionally active estrogens. Hydrolysis transforms dehydroepiandrosterone sulfate (DHEA-S), estrone sulfate (E1S), the most abundant circulating estrogen metabolite, and estradiol sulfate (E2S) into their bioactive metabolites (DHEA, E1 and E2, respectively). Despite its potential pivotal roles in local estrogen formation, data on metabolic activity of this enzyme are not very conclusive. An analysis of 27 peritoneal endometriosis implants showed lower overall STS activity in ectopic endometrium than in eutopic endometrium (54). The authors attributed this to the relatively lower enzyme activity levels in endometriotic lesions from patients with minimal to mild disease, and indeed, with further analysis, they observed that STS activity in endometriosis implants correlates with the severity of this disease, and a significantly higher activity of STS was found in patients with moderate to severe disease with respect to controls, indicating that women with severe endometriosis may be particularly amenable to STS inhibitor therapy. However, Delvoux et al. did not find a difference in terms of STS enzyme activity between ectopic and eutopic endometrial tissue despite the analysis being provided in women affected by moderate to severe endometriosis from all three types of endometriosis (23).

### Sulfatase expression on human tissue

To date, evidence on the expression of *STS* in endometriosis has remained relatively contradictory (**Table 5**). Only two studies evaluated STS at the protein level (41, 45), reporting no difference between cases and controls. STS mRNA expression was greater in superficial, ovarian and deep-infiltrating lesions (no significant differences were found between these two types of lesions) of endometriosis samples than in eutopic endometrium of subjects without endometriosis (43, 45, 56). Conflicting findings have been published by from Dassen et al., that, evaluated 14 women with rectovaginal endometriosis and did not observe any differences between STS mRNA levels of endometriosis and healthy tissues (P, 0.05) (41).

**Table 5:** STS and SULT1E1 mRNA, protein expression in endometriotic tissue.

	Patient with endometriosis	Patient characteristics	Method	STS expression (compared to healthy endometrial tissue)		SULT1E1 expression (compared to healthy endometrial tissue)	
				mRNA	Protein (Ab)	mRNA	Protein (Ab)
Hudelist et al 2007 (18)	35	Mixed lesions. Mild to moderate endometriosis	QPCR IHC	-	-	≈	≈ (NeoMarkers)
Dassen et al 2007 (41)	14	Rectovaginal endometriosis	QPCR IHC	≈	≈ (Pineda)	↑	-
Smuc et al 2007 (42)	16	Endometrioma (stage III, IV)	QPCR	↑	-	≈	-
Colette et al 2013 (45)	79	Mixed lesions	Q-PCR IHC	↑ Rectovaginal lesion	≈ (Atlas)	≈	-
Hevir et al 2013 (55)	31	Ovarian endometriomas	QPCR	-	-	↓	-
Piccinato et al 2016 (56)	62	Peritoneal Deep lesions	Q-PCR	↑	-	↑	-

Ab, Antibodies: ASRM, American Society for Reproductive Medicine; HPLC high performance liquid chromatography; IHC, immunohistochemistry; Q-PCR, Quantitative-Polymerase chain reaction; STS, steroid sulfatase, ↑/↓ statistically significant results: ≈ , no difference.

### Sulfotransferase expression in human tissue

Estrone sulfotransferase (SULT1E1), in contrast to STS, antagonizes the action of STS by sulfating estrone into esterone sulfate, thus converting estrogens into less active metabolites. However, the expression of this enzyme in endometriosis lesions demonstrated a contradictory pattern. The evaluation of patients with mild and moderate endometriosis shows no significant differences between the expression levels of SULT1E1 protein in uterine and ectopic samples in comparison to the endometrium of healthy women (18). In endometrioma samples, Hevir et al. found that SULT1E1 mRNA levels were significantly decreased compared to controls (55), whereas Smuc et al. registered no significant difference in its expression (42). Colette et al. showed that in rectovaginal endometriosis, although no difference was encountered in SULT1E1 mRNA expression, there was a high ratio between STS and SULT1E1, giving rise to the view that in endometriosis lesions, the sulfatase pathway is overactive (45). Interestingly, two studies reported a higher expression of SULT1E1 in endometriotic lesions than in normal endometrium: rectovaginal endometriosis lesions (41) and superficial peritoneal lesions (56) showed higher expression of EST compared to the control and had a positive correlation with STS expression. If STS abounds over SULT1E1, the increased net production of estradiol in endometriosis is the directed consequence; in this way, sufficient sulfated estrogens can be continuously hydrolyzed (desulfated) and sulfated *in situ*, maintaining a highly local estrogenic milieu.

### Sulfatase inhibitors

Based on the aforementioned data, STS can be considered an attractive molecular target with potential therapeutic value in endometriosis, and targeting this enzyme may benefit patients with resistance to other hormonal treatments. Purohit et al. tested a recent irreversible STS inhibitor, 667COUMATE (also called Irosustat), which was already assessed in postmenopausal women with metastatic breast cancer. It proved to be very effective at inhibiting STS activity in endometriotic cell lysates, reducing enzyme activity by 99% in both eutopic and ectopic endometrial tissue samples (54). Another inhibitor, estradiol-3-O-sufamate (E2MATE), also encoding PGL2001, was proven to effectively inhibit STS activity when tested *in vitro* on endometrial fragments of ten patients affected by benign pathologic conditions other than endometriosis (STS activity inhibition after 24 h of culture: 66.5 + 10.3%, P, 0.001). Endometriosis was then induced in mice to evaluate the inhibition *in vivo* of this enzyme. After twenty-one days of therapy, lesion sizes were found to be significantly decreased (control mice: 44.5 ±30.2 mm2; 1 mg/kg-treated mice: 26.3±20.1 mm2; 0.5 mg/kg treated mice: 22.8±15.3 mm2) (57). As an additional benefit, progesterone (PR) expression in endometriotic lesions was found to be increased, and the absence of an effect on circulating estradiol levels opens up new perspectives in endometriosis treatment. E2MATE was then evaluated in a phase I double-blind study (58).Given that the majority of estrogens are produced in the ovaries, the authors focused their evaluation on a combination STS-I plus progestin in order to reduce both the local and ovarian estrogen production. Twenty-four healthy volunteer women were randomized to E2MATE (4 mg/week), NETA or the combination E2MATE+NETA. Treatment lasted 4 weeks with a 12-week follow-up. E2MATE associated with NETA showed a synergistic effect: the mean percentage of STS inhibition in the endometrium was 91% and 96% in the PGL2001 and PGL2001 plus NETA groups, respectively, compared to 42% in the NETA group, and due to its potent irreversible binding and long half-life one month after stopping treatment, the percentage inhibition remained high at 88% and 93% in the PGL2001 and PGL2001 plus NETA groups, respectively, with no inhibition seen in the NETA group. Treatment was well tolerated, with no relevant differences between the treatment regimens in terms of adverse events, and no impact on circulating estradiol levels was registered compared to the NETA groups. E2MATE and NETA have been further studied in endometriosis patients in a phase II study (NCT01631981, available at: https://clinicaltrials.gov/ct2/show/NCT01631981), although at present, no results are publicly available. An interesting recent development is the establishment of multiple designed ligands that effectively inhibit both STS and HSD17B1 (59).Such dual inhibitors can further decrease intracellular E2 levels more efficiently than selective inhibitors of HSD17B1 and may therefore be a superior therapeutic strategy for endometriosis.

## Effect of current hormonal treatments on intracrinology regulation

Current first-line hormonal treatments in endometriosis (oral contraceptives and progestins) were originally developed using the normal endometrium as the main experimental tissue to investigate their reproductive effects and were adopted only afterwards for the treatment of endometriosis-associated pain. There are several patients for whom current first-line hormonal treatments for endometriosis do not provide enough or a sustained solution to pain. A recent review demonstrated that the median proportion of women with no decrease in pain was 11% to 19%; when the therapy ended, 5% to 59% had persistent pain; and in the follow-up, 17% to 34% felt recurrence of pain symptoms (60). The recent evidence that women, despite the hypogonatrophic effect obtained from combined oral contraceptives (COCs), have increased hormone levels in endometriosis implants compared to controls (61) highlights the crucial role of intracrinology as a mechanism of endometriosis development and drug resistance. There are only a few available research studies that have investigated the influence of the commonly used therapy against the enzymes involved in the intracrinology of the endometriosis. Few are based on progestins, and no research studies have investigated the effects of COC. Most studies, in addition, have been conducted *in vitro* using immortalized cells, limiting a realistic interpretation of the results. Dienogest (DNG), a synthetic progestin largely employed for therapy, has received the greatest attention. DNG has been shown to repress aromatase expression in human immortalized endometrial epithelial cells and primary cultured endometriotic stromal cells (SCs) (62, 63). Moreover, DNG has been shown to inhibit HSD17B1 expression and enzymes in cultured ovarian endometrioma cells, whereas no effect has been demonstrated on HSD17B2, HSD17B7, HSD17B12, steroid sulfatase (STS), and estrogen sulfotransferase (EST) activity (64). There are few details with respect to GnRH agonist and antagonist effects on endometrial intracrinology. While GnRH agonists have been shown to decrease serum E2 levels by approximately 97%, intracrinological changes in endometriosis lesions are not known. Even if GnRH agonists are responsible for decreasing tissue inflammation and angiogenesis and increasing apoptosis in endometriosis (65), 14% (0-20%) of patients did not show improvement of symptoms, and nearly one-third of patients who received GnRH analog treatment postsurgery experienced pain symptoms when medical treatment ended (60). Interestingly, one-year therapy with a GnRH analog decreases the adrenal DHEA-S combination by only 16%, leaving open the possibility of its metabolism in peripheral tissue and eventually inducing resistance to treatments (66). GnRH agonists have been shown to reduce aromatase cytochrome P450 expression in at least eutopic endometrium from patients with endometriosis (67) and hinder E1 sulfatase expression in endometrioma (68). Recently, GnRH antagonists have been augmented in the armamentarium of gynecologists to cure endometriosis and resolve the side effects of GnRH agonists based on the “estrogen threshold hypothesis,” where estrogen may be regulated to a level that is enough to reduce pain without causing clinical hypoestrogenic effects. Elagolix did not fully repress ovulation at doses of 150 and 200 mg/day, 56% of women had proliferative endometrium after 6 months of therapy at a dose of 150 mg, and 61% had normal dormant or least stimulated endometrium at a dose of 200 mg. Even if the majority of patients were satisfied with this therapy, as many as 40% of patients indicated unsatisfactory improvement of pain symptoms (69). On the basis of the complexity of the intracrinology of endometriosis and the fact that GnRH antagonists have no direct effect on the endometrium, we can then hypothesize that the intracrine features of endometriosis may represent a mechanism creating an incomplete response to symptoms of pain in 30-35% of patients

## Discussion

The study of intracrinology in endometriosis highlights important limitations in the current knowledge of the disease, from its developmental and initial phases to the macroscopic appearance of advanced deep nodules. It is clear that the current macroscopic classification is insufficient to properly characterize heterogeneous lesions. A closer look at endocrine aspects of lesions may shed new light on disease features, enabling precision medicine in endometriosis care. However, current methodological limitations (i.e., number of patients enrolled in the studies) or appropriateness of investigations (i.e., contamination of endometrioma cysts with ovarian cortex) has limited the implementation of endocrine phenotyping in daily practice. Furthermore, the accurate evaluation of the expression of these enzymes is a complex task; for instance, concerning aromatase protein expression, some authors argued that what was believed to be aromatase protein in a previous study was mainly endogenous biotic labeling or iron deposits (20). Even if various enzymatic pathways are aberrantly regulated in endometriosis, the recognition of which enzymatic pathways are more critical for promoting the hyperestrogenic environment is less clear. This is important for the development of new drugs aimed at decreasing local estrogenic activity with a minimal effect on gonadal activity.

### Strengths and limitations

The strength of this review lies in the evaluation of intracrinology of endometriosis from a clinical prospective. The main limitation was that heterogeneity of the studies included and a lack a particular model to investigate the role of local modulation of these enzymatic pathways.

### Conclusion and new perspectives

Unbalanced intracrinology is a critical feature of endometriosis implants and a complex mechanism that supports local hyperestrogenism partially independent from gonadal function. This has far-reaching implications in clinical practice, since all available therapies induce a reduction in gonadal activity as main mechanism of action. Recently, the development of harmonization initiatives, such as EPHect, Endometriosis Phenome and Biobanking Harmonisation Project, has represented a new systematic approach to stratify predefined outcomes in endometriosis research with family history, symptoms, clinical examination, dynamic imaging/pain reporting, surgical staging, and systemic or tissue biomarkers. From a clinical perspective, current knowledge of intracrinology in endometriosis in the actual classification of the disease has identified that endometriosis lesions on the ovary are likely the most endocrine active and responsive to steroids. As a result, they are characterized by a higher incidence of recurrence following surgical excision. In addition, enzymatic pathways expressed in endometriosis are likely consequences of epigenetic changes and inflammation signals. Again, a closer look at intracrinology could facilitate lesion phenotyping and estimate the aggressiveness of the disease. However, current methodological limitations and heterogeneity in the evaluation of mRNA and protein expression make it hard to draw definitive conclusions. In certain cases, contradictory results can be explained by the close proximity of healthy tissue to the endometriotic lesion, influencing the results obtained by the whole tissue specimens and highlighting the need for a careful histopathological characterization of the specimens studied (laser capture microdissection may therefore be envisaged to fully isolate endometriotic glands). Moreover, a large number of studies miss correlations with the severity of symptoms, stage and localization of disease, making it difficult to estimate which patient can benefit the best from future targeted enzyme therapy. Although AIs are not realistically useful in clinical practice, intracrinology offers interesting new drug targets that can incorporate many of the above ambitious features. Some molecules are already in the pipeline of the pharma industry in the next 10 years. In conclusion, intracrinology of endometriosis is relevant in clinical practice as a major main endometriosis developmental feature, a basis for phonotypical characterization, a potential mechanism of drug resistance and a source of new therapeutic targets.

## Data availability statement

The raw data supporting the conclusions of this article will be made available by the authors, without undue reservation.

## Author contributions

AM and NP undertook the searches, data extraction and drafted the manuscript. NP, AM, PG, PD, and AR participated in data analysis and interpretation, preparation of. the manuscript and critically revising the paper. NP and AR conceived the idea of the manuscript. All authors approved the final version of the manuscript.

## Funding

Open access funding was provided by the University of Geneva.

## Conflict of interest

The authors declare that the research was conducted in the absence of any commercial or financial relationships that could be construed as a potential conflict of interest.

## Publisher’s note

All claims expressed in this article are solely those of the authors and do not necessarily represent those of their affiliated organizations, or those of the publisher, the editors and the reviewers. Any product that may be evaluated in this article, or claim that may be made by its manufacturer, is not guaranteed or endorsed by the publisher.

## Abbreviations

AIs, Aromatase inhibitors; HSD17B, 17β-hydroxysteroid dehydrogenase; STS, Steroid sulfatase; SULT1E1, Estrogen sulfotransferase; DHEA, Dehydroepiandrosterone; DNG, Dienogest; T, testosterone; E1, estrone; E2, estrdiol; A4, Androstenedione; COX-2, cyclooxygenase-2; PGE2, Prostaglandin E2; COCs, combined oral contraceptives; GnRHa, gonadotropin-releasing hormone agonist; NETA, norethisterone acetate; GnRHa, gonadotropin-releasing hormone agonist; IVR, Intra vaginal ring; AKR1C3, 17β-hydroxysteroid dehydrogenase type 5.
