# Erratum: Is intracrinology of endometriosis relevant in clinical practice? A systematic review on estrogen metabolism

**DOI:** 10.3389/fendo.2023.1177346

**Published:** 2023-03-10

**Authors:** Frontiers Production Office

**Affiliations:** Frontiers Media SA, Lausanne, Switzerland

**Keywords:** endometriosis, estrogen, metabolism, pelvic pain, endometrium

An omission to the funding section of the original article was made in error. The following sentence has been added: “Open access funding was provided by the University of Geneva”.

The original version of this article has been updated.

